# Preparation and Compatibility Evaluation of Polypropylene/High Density Polyethylene Polyblends

**DOI:** 10.3390/ma8125496

**Published:** 2015-12-17

**Authors:** Jia-Horng Lin, Yi-Jun Pan, Chi-Fan Liu, Chien-Lin Huang, Chien-Teng Hsieh, Chih-Kuang Chen, Zheng-Ian Lin, Ching-Wen Lou

**Affiliations:** 1 Laboratory of Fiber Application and Manufacturing, Department of Fiber and Composite Materials, Feng Chia University, Taichung City 40724, Taiwan; jhlin@fcu.edu.tw (J.-H.L.); chengyen0624@gmail.com (Z.-I.L.); 2 School of Chinese Medicine, China Medical University, Taichung City 40402, Taiwan; 3 Department of Fashion Design, Asia University, Taichung City 41354, Taiwan; 4 Department of Materials and Textiles, Oriental Institute of Technology, New Taipei City 22061, Taiwan; fc003@mail.oit.edu.tw; 5 Office of Physical Education and Sports Affairs, Feng Chia University, Taichung 407, Taiwan; cfliu@fcuoa.fcu.edu.tw; 6 Department of Fiber and Composite Materials, Feng Chia University, Taichung City 40724, Taiwan; clhuang@mail.fcu.edu.tw; 7 Department of Fashion Design and Merchandising, Shih Chien University Kaohsiung Campus, Kaohsiung City 84550, Taiwan; edo@mail.kh.usc.edu.tw; 8 The Polymeric Biomaterials Laboratory, Department of Fiber and Composite Materials, Feng Chia University, Taichung City 40724, Taiwan; chihkchen@fcu.edu.tw; 9 Institute of Biomedical Engineering and Materials Science, Central Taiwan University of Science and Technology, Taichung City 40601, Taiwan

**Keywords:** polypropylene (PP), high density polyethylene (HDPE), polyblend, melt-blending, compatibility

## Abstract

This study proposes melt-blending polypropylene (PP) and high density polyethylene (HDPE) that have a similar melt flow index (MFI) to form PP/HDPE polyblends. The influence of the content of HDPE on the properties and compatibility of polyblends is examined by using a tensile test, flexural test, Izod impact test, scanning electron microscopy (SEM), Fourier transform infrared spectroscopy (FTIR), differential scanning calorimetry (DSC), polarized light microscopy (PLM), and X-ray diffraction (XRD). The SEM results show that PP and HDPE are incompatible polymers with PP being a continuous phase and HDPE being a dispersed phase. The FTIR results show that the combination of HDPE does not influence the chemical structure of PP, indicating that the polyblends are made of a physical blending. The DSC and XRD results show that PP and HDPE are not compatible, and the combination of HDPE is not correlated with the crystalline structure and stability of PP. The PLM results show that the combination of HDPE causes stacking and incompatibility between HDPE and PP spherulites, and PP thus has incomplete spherulite morphology and a smaller spherulite size. However, according to mechanical property test results, the combination of HDPE improves the impact strength of PP.

## 1. Introduction

Polyblends are a product by melt-blending or solvent-blending two or more polymers [1,2,3,4]. The mechanical or physical properties of polyblends depend on the phase morphology, action between continuous and dispersed phase, and the component ratios [5]. In terms of processing technique, phase morphology relies on the processing technique, including extrusion, injection molding, and manufacturing conditions, such as temperature and shear force.

For real applications, polyblends are mostly made by physical blending of melt processing, which blends various polymers on an extruder, compounder and mixer. Melt processing does not require solvents to attain polyblends with high dispersion; the essential factors being meticulous temperature control and operation duration. However, attention to the thermal degradation caused by high shear force and high temperature is required [6,7,8,9].

PP is one of the most commonly used polymers, and has good mechanical properties, heat resistance, low cost, ease of processing, and full recyclability. Its biggest drawback is low impact strength, which can be improved by a toughening modification. Therefore, a blending method, the most efficient and easy, has been widely used. For a toughening modification, other thermoplastics or elastomers are used as modifiers to blend with PP in order to increase its toughness [10,11,12,13,14,15]. This study uses HDPE, which has a similar structure to PP, ease of processing, low cost, and impact resistance, to improve the impact strength of PP.

Apart from PP-based and PE-based composites, PP/PE polyblends and composites have also been commonly studied [1,16,17,18,19]. In addition, the crystallization behavior, phase morphology, and processing technique are crucial to the structure and properties of the PP/PE polyblends. Souza *et al.* examined the influence of processing temperature and the content of HDPE on the interfacial tension of the PP/HDPE polyblends, and found that the interfacial tension is inversely proportional to the two conditions [20]. Li *et al.* examined the blends containing PP and PET at various densities, and concluded that the LLDPE/PP blends possess the optimal compatiblity, and their dispersed phase determines the mechanical properties. On the other hand, the dispersed phase of the VLDPE/PP blends significantly decreases as a result of thermal treatment [21]. According to previous studies, the compatibility of polyblends depends on the processing temperature, polymer structure, and blending ratios [20,21,22]. In addition, it was also indicated that the combination of two polymers that were formed beforehand with similar conditions or possess similar physical properties contributes to greater mechanical properties of the compounds [23,24,25]. There are few studies examining the relationship between the melt flow index and polymer blends. Jose *et al.* combined PP and HDPE that have a dramatic range of their melt flow index (MFI), and found a significant phase separation between these two materials that decreases the mechanical properties of the compounds [22]. Therefore, this study combines PP and HDPE that have similar MFI in order to comprehensively compare the improvement of HDPE on the compatibility between HDPE and PP, as well as the impact strength of the PP/HDPE polyblends. In addition to mechanical property tests, different tests are also performed for different purposes that are highly correlated with the compatibility as follows: crystallization temperature and rate (DSC), spherulite size (PLM), crystal structure (XRD), phase separation (SEM), and molecular structure (FTIR). This study contributes a helpful manufacturing for combining HDPE and PP that have a similar melt flow index, and successfully decreases the phase separation between two materials, and thereby increases the compact strength of PP.

## 2. Results and Discussion

### 2.1. Mechanical Properties of PP/HDPE Polyblends

**Figure 1:**
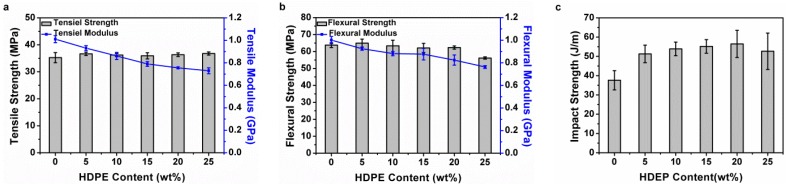
(**a**) Tensile properties, (**b**) flexural properties, and (**c**) impact strength of the polypropylene/high density polyethylene (PP/HDPE) polyblends.

**Figure 2:**
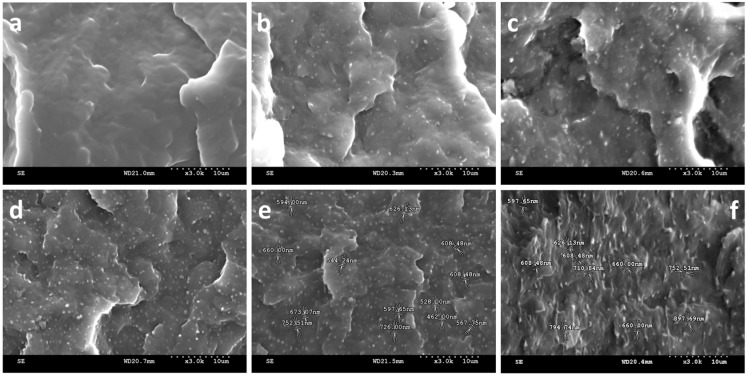
Scanning electron microscopy (SEM) images (3000×) of (**a**) is the fractured PP matrix, and the fractured PP/HDPE polyblends, which contain (**b**) 5 wt %; (**c)** 10 wt %; (**d**) 15 wt %; (**e**) 20 wt %, and (**f**) 25 wt % of HDPE.

Figure 1a shows that the tensile strength of PP matrices is 35 MPa, and that of PP/HDPE polyblends is 35 MPa when the content of HDPE is 25 wt %. Such a result indicates that the combination of HDPE does not influence the tensile strength. Figure 1b shows that the flexural strength of PP is 63 MPa, and that of PP/HDPE polyblends with 20 wt % of HDPE is 60 MPa. However, when HDPE is 25 wt %, the flexural strength of the polyblends declines. This result is due to the softness and toughness of the HDPE, which are both good, thus influencing the flexural strength of the PP matrices. In addition, Figure 1a,b indicate that a high HDPE content causes the tensile modulus and flexural modulus of the PP/HDPE polyblends to decrease, which is ascribed to two reasons. One reason is the phase separation of HDPE, which gives rise to HDPE particles that serve as the nucleating agent for PP. The spherulite size of PP is thus decreased, which is adverse to the crystallinity of PP. The tensile modulus and flexural modulus are decreased when PP has a low crystallinity. The other reason is that HDPE has a lower tensile modulus and flexural modulus in comparison to PP. As a result, the higher the HDPE content, the lower the tensile modulus and flexural modulus the PP/HDPE polyblends have. Figure 1c shows that the impact strength of PP matrices is 38 J/m, and when combined with 20 wt % HDPE, the impact strength increases to 56 J/m. As HDPE is distributed in PP matrices in the forms of particles (Figure 2), and when impact force is exerted, the particles exhibit stress concentration and also exhibit plastic deformation to dissipate the impact energy, thereby reinforcing the impact strength of the polyblends [24,26]. Another factor is the structure of HDPE, which are long polymer chains and thus have a considerable softness. The impact strength of the polyblends increases as a result of the content of HDPE.

### 2.2. SEM of PP/HDPE Polyblends

Figure 2a shows that the fractured surface of PP matrices is smoother, featuring a brittle fracture. Figure 2b–f shows that the fractured surface of the PP/HDPE is ragged, which features a toughness fracture. A previous study indicated that the key point to phase morphology composed of the continuous phase and dispersed phase is determined by the ratio of the components, variation in the viscosity, and melt-blending conditions [27]. 

PP has a greater melt index than that of HDPE, indicating that HDPE has a greater viscosity. The material with a greater viscosity is not easily sheared and separated, and thus is commonly present in the form of a dispersed phase. Conversely, the low viscous material has an even form after the melt-blending, and is present in a continuous phase [28]. In addition, the formation of HDPE particles results in an interface between HDPE and PP, which results in a phase separation in PP/HDPE polyblends that is in relation to their mechanical properties [29]. Other influential factors reported in previous studies include interface adhesion, dispersion, and distribution of the dispersed phase [23,25,30,31]. The combination of two components with a close MFI causes a good dispersion and smaller average size of the dispersed phase in the continuous phase. PP and HDPE have a similar MFI, and HDPE are transformed into particles of a smaller size (0.4–0.7 μm) and are evenly distributed in the continuous phase (PP). The contact area between HDPE and PP also increases.

Figure 2e shows that a 20 wt % of HDPE results in a greater amount of HDPE particles. The cracks occur as a result of the exerted strain, the HDPE particles that are in a dispersed phase bridge each crack to prevent the cracks from distending. Meanwhile, these HDPE particles also absorb a great deal of energy caused by an external force, and thereby augmenting the impact strength of the polyblends [24,26]. These results are consistent with that shown in Figure 1c. However, excessive HDPE content (25 wt %) increases the level of phase separation of HDPE, and leads to the presence of interfaces between PP and HDPE as indicated in the circle in Figure 2f. Although the phase separation of HDPE particles can also be found while HDPE content is 15 wt %, the presence of HDPE particles is favorable to the impact strength of the polyblends. However, the occurring of interface caused by excessive HDPE content is adverse to tensile modulus and flexural modulus of the polyblends.

### 2.3. Chemical Structure of PP/HDPE Polyblends

**Figure 3:**
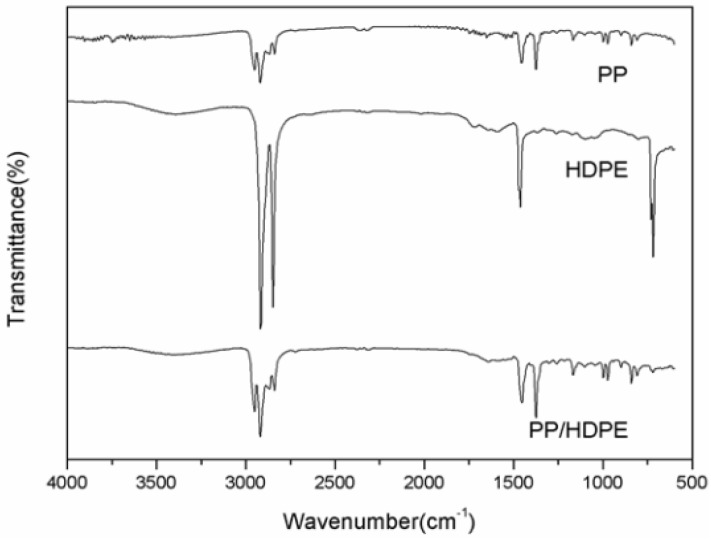
Fourier transform infrared spectroscopy (FTIR) spectra of of PP/HDPE polyblends.

An FTIR examines the chemical structures of PP/HDPE polyblends. Figure 3 shows the frequency range as related to different vibration types of methyl (PP/HDPE): the stretching vibration of –C–H is 2985–2810 cm^−1^ (PP) and that of –CH_2_ is 2950–2850 cm^−1^ (HDPE); the bending vibrations of –CH_2_ and –CH_3_ are 1475–1440 cm^−1^ (PP) and 1380–1370 cm^−1^ (PP), respectively, and that of –CH_2_ is 1470–1460 cm^−1^ (HDPE); and the rocking vibration of −CH_2_ is 730–700 cm^−1^ (HDPE). According to the FTIR spectra, all the peaks of the PP/HDPE polyblends are in conformity with those of PP the matrices. This indicates that the main chemical structure of the polyblends is PP that is not in relation to the combination of HDPE [32]. This is ascribed to HDPE and PP that are both polyolefins polymers and are not compatible. In addition, SEM images of PP/HDPE polyblends (Figure 2) also indicates a significant presence of phase separation between PP and HDPE, which proves that the polyblends are formed via a physical blending.

### 2.4. Non-Isothermal Crystallization and Melting Behaviors of PP/HDPE Polyblends

**Figure 4:**
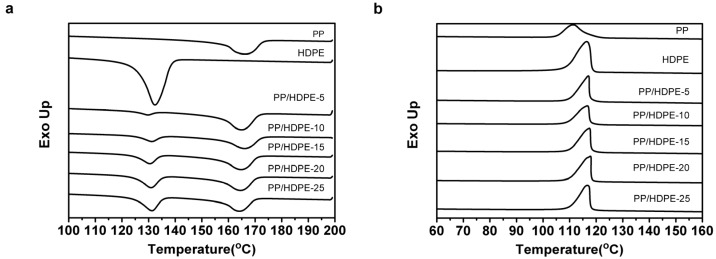
(**a**) Melting peak and (**b**) crystallization peak characterized in the differential scanning calorimetry (DSC) curve of PP/HDPE polyblends.

Table 1 shows that the melting temperatures of PP and HDPE are 166.01 and 132.39 °C, respectively. Figure 4a shows that PP/HDPE polyblends possess two melting peaks, indicating that PP and HDPE co-exist. Furthermore, from Table 1, even though the polyblends are composed of various contents of HDPE and PP, the melting temperatures of PP and HDPE do not distinctly fluctuate, which proves that the combination of HDPE does not influence the crystallinity and stability of PP and HDPE in the polyblends.

Table 1 shows that the crystallization temperatures of PP and HDPE are 111.38 and 116.48 °C, respectively, and both of the temperatures are different. However, the crystallization orders of PP and HDPE are quite close when the non-isothermal temperature goes down. Their crystallization peaks cannot be distinguished in Figure 4b, which is due to the quick crystallinity rate of HDPE. HDPE has a faster crystallization than PP does. Moreover, the combination of HDPE also accelerates the heterogeneous nucleating of PP in the polyblends. As a result, PP can also have a quick crystallization, and thus the crystallization peaks of PP and HDPE cannot be distinguished from one other.

**Table 1:** Differential scanning calorimetry (DSC) data of polypropylene/high density polyethylene (PP/HDPE) polyblends.

Sample	∆*H*_m_ (J/g) ^a^	*T*_m_ (°C) ^a^	*T*_c_ (°C) ^a^	*X*_c_ (%) ^a^	∆*H*_m_ (J/g) ^b^	*T*_m_ (°C) ^b^	*X*_c_ (%) ^b^
PP	111.0	166.0	111.3	53.1	-	-	-
HDPE	-	-	116.5	-	210.3	132.4	75.1
PP/HDPE-5 wt %	126.8	164.8	117.1	63.9	8.2	129.7	58.5
PP/HDPE-10 wt %	106.1	166.0	116.7	56.4	19.6	131.0	70.0
PP/HDPE-15 wt %	121.3	164.8	117.4	68.3	31.7	130.5	75.5
PP/HDPE-20 wt %	80.2	164.6	117.8	63.1	44.9	130.9	80.2
PP/HDPE-25 wt %	70.9	164.1	116.7	45.2	50.3	132.3	71.8

**^a^** Refers to the specifications of PP while **^b^** refers to the specifications of HDPE.

### 2.5. PLM Observation of PP/HDPE Polyblends

**Figure 5:**
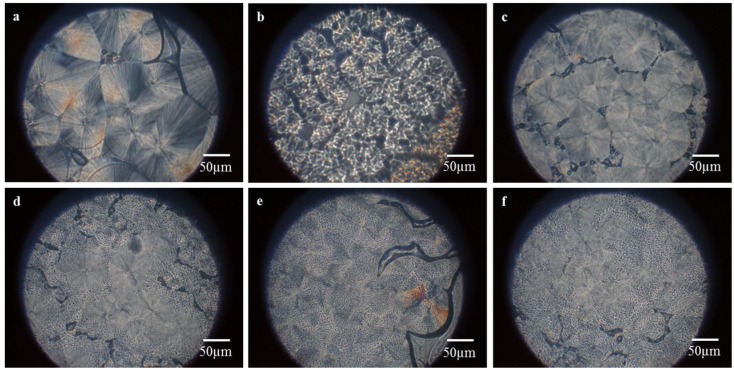
Polarized light microscopy (PLM) images of (**a**) pure PP film; (**b**) HDPE film, and PP/HDPE polyblends, which contain (**c**) 5 wt %; (**d**) 10 wt %; (**e**) 15 wt %, and (**f**) 20 wt % of HDPE.

Figure 5a shows that PP has large spherical spherulites with a Maltese cross on their surface. Figure 5b shows that HDPE has spherulites with a complicated morphology, in which Maltese crosses are overlapped with concentric rings, and thus are called ringed spherulites. Figure 5c–f shows that an increasing HDPE prevents the HDPE spherulites in adjacence from growing, and as a result, the spherulites cannot be formed into a complete form. On top of the stack of PP spherulites and HDPE spherulites, the PP spherulites end up being incomplete and distinctly smaller [18,19]. PP has a greater size of spherulites, which are not bonded well and thus PP has low impact strength. Adding HDPE decreases the size of spherulites and increases the contact area of spherulites, which in turn contributes to the impact strength of PP/HDPE polyblends, as seen in Figure 1c.

### 2.6. Structure Characterization of PP/HDPE Polyblends

**Figure 6:**
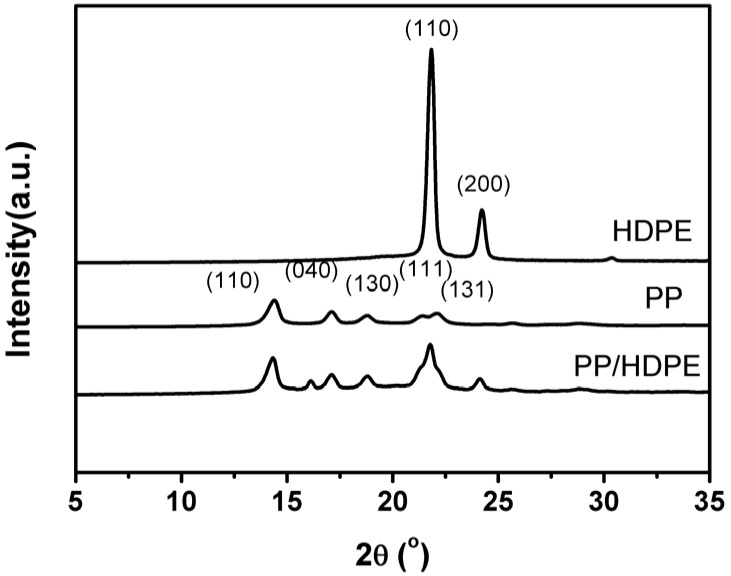
X-ray diffraction (XRD) curve of PP/HDPE polyblends.

The crystal structure of PP/HDPE polyblends is analyzed by XRD. Figure 6 shows that when 2θ is between 10° and 25°, PP has five diffraction peaks, which consist of PP’s typical α-form. The array for 2θ with corresponding crystalline lattices are 14.28° (110), 17.14° (040), 18.92° (130), 21.40° (111), and 22.20° (041) [33]. The 2θ with corresponding crystalline lattices for HDPE are 21.6° (110) and 23.9° (200) being composed of orthorhombic crystals [34,35]. PP/HDPE polyblends have identical crystalline lattices as those of PP, except for 2θ = 21.8°.

The XRD curve shown in Figure 6 and the DSC curve in Figure 5 also indicate that the combination of HDPE does not affect the main chemical structure of PP. The crystal structure of PP does not change as a result of the combination of HDPE, and has an α-foam structure [33]. Therefore, the crystal stability of PP is not correlated with the presence of HDPE.

## 3. Experimental Section

### 3.1. Materials

PP (YUNGSOX 1080; Formosa Plastics Corporation, Taipei, Taiwan) is a homopolymer. HDPE (8050, injection molding grade) is purchased from Formosa Plastics Corporation, and Table 2 summarizes its physical properties.

**Table 2:** Physical properties of the two polymers.

Materials	Density (g/cm^3^)	Melt Index (g/10 min)	Molecular Weight
PP	0.900	10 (230 °C/5 Kg measured)	80,000–90,000
HDPE	0.960	6 (230 °C/5 Kg measured)	75,000–80,000

### 3.2. Preparation of Polyblends

HDPE (0, 5, 10, 15, 20 and 25 wt %) and PP are blended and dried in an oven at 60 °C for 8 h to remove moisture, after which they are made into polyblend pellets at a screw speed of 24 rpm on a single screw extruder (SEVC-45, Re-Plast Extruder Corp., Miaoli, Taiwan); the temperatures of the three barrels are 190, 200 and 210 °C, and the temperature of the die is 220 °C. The pellets are dried at 60 °C for 8 h, and then made into test samples on an injection machine (Ve-80, Victor Taichung Machinery Works Co., Ltd., Taichung, Taiwan); the temperatures of the three barrels are 190, 200 and 210 °C, and the temperature of the nozzle is 220 °C.

### 3.3. Measurements and Characterization

#### 3.3.1. Mechanical Properties

Tensile strength of the PP/HDPE polyblends is tested with an Instron 5566 (Instron, Canton, MA, USA), as specified in ASTM D638-14 [36]. Samples are prepared according to ASTM D638 Type IV. The distance between the two clamps is 25 mm, the test speed is 50 mm/min, and five samples of each specification are taken.

An Instron 5566 (Instron) measures the flexural strength of the PP/HDPE polyblends, as specified in ASTM D790-10 [37]. The test speed is 2 mm/min, and the support span is 50 mm. Measuring 127 mm × 12.7 mm × 3.2 mm, five samples of each specification are taken. The flexural strength is calculated with results being calculated with the following equation:

(1) $$\sigma_{\text{fmax}}=\frac{3PL}{2bd^{2}}$$

where σ_fmax_ is the flexural strength (MPa), *P* is the load (N), *L* is the support span (mm), *b* is the width of sample width (mm), and *d* is the sample thickness (mm).

The impact strength of the PP/HDPE polyblends is measured with an Izod impact strength tester (CPI, ATLAS, Mount Prospect, IL, US), as specified in ASTM D256-10e1 [38]. Five samples of each specification are taken, and samples measuring 63.5 mm × 12.7 mm × 3.2 mm have a V-shape cut of 45° and a depth of 0.25 mm.

#### 3.3.2. Scanning Electron Microscopy (SEM)

The fractured samples collected from the impact strength test are observed at an operation voltage of 15 kV with a scanning electron microscope (S3000N, Hitachi, Tokyo, Japan), with their surface being coated with a thin layer of gold and with them affixed to the sample holder. The fractography are compared to the results of the impact strength test.

#### 3.3.3. Fourier Transform Infrared Spectroscopy (FTIR)

The chemical structure of PP/HDPE polyblends is analyzed with an FTIR (IRAffinity-1, Shimadzu Corporation, Kyoto, Japan). The spectra are recorded in the range of 4000 to 600 cm^−1^. The air, which serves as background, is scanned in advance, after which samples are scanned with the same scanning range for further analyses.

#### 3.3.4. Differential Scanning Calorimetry (DSC)

**Figure 7:**
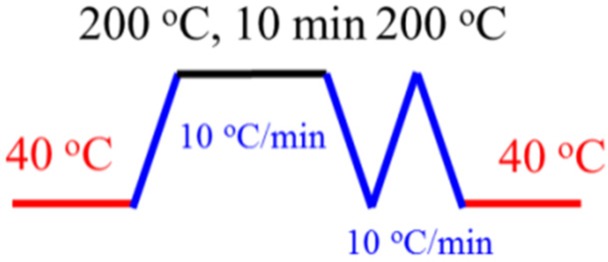
Differential scanning calorimetry (DSC) controlling curve.

The crystallization behavior of PP/HDPE polyblends is analyzed by a DSC (Q200, TA Instruments, New Castle, DE, USA). An amount of 8 to 10 mg of samples is placed in the DSC which is then heated from 40 to 200 °C at 10 °C/min increments and kept at 200 °C for 10 min, and finally cooled from 200 to 40 °C at 10 °C/min increments. The heating and cooling process, which is illustrated as Figure 7, is repeated one more time in order to delete the thermal history of the material.

The degree of crystallinity of PP and HDPE in the PP/HDPE polyblends is calculated by the following equation:

(2) $$X_{\text{C}}\left(\text{\%}\right)=\frac{∆H_{\text{m}}}{\left(1-\emptyset \right){∆H}_{\text{m}}^{{}^{\circ}}}\times 100\text{\%}$$

where *X*_c_ is the crystallinity, ∆*H*_m_ is the apparent enthalpy of crystallization, $∆H_{\text{m}}^{{}^{\circ}}$ is the enthalpy corresponding to the melting of 100% crystalline PP and HDPE, and ∅ is the weight fraction of the matter. According to previous studies, the ∆*H*_m_ of PP is 209 J/g and that of HDPE is 280 J/g [39,40].

#### 3.3.5. Polarized Light Microscopy (PLM)

A polarized light microscope (BX51, Olympus, Tokyo, Japan) is used to observe the spherulite morphology of PP/HDPE polyblends. A few samples are placed on the glass slide and melted at 200 °C to form films. Afterwards, the temperature is decreased to 130 °C at 10 °C/min increments, and remains constant in order to observe the spherulite morphology of the samples.

#### 3.3.6. X-ray Diffraction (XRD)

The crystal structure of PP/HDPE polyblends is examined by an XRD (MXP3, Mac Science, Yokohama, Japan). Samples are first melt-compressed into 1 cm × 1 cm squares, and then scanned within a range of 5°–35° with Cu Kα radiation at 40 kV and 30 mA. The scanning rate is 2°/min and λ = 0.154 nm.

## 4. Conclusions

This study successfully compounds two polyolefins with similar MFI and improves their dispersion. The test results show that a 20 wt % of HDPE maintains a certain level of tensile strength and flexural strength, and increases the impact strength of PP/HDPE polyblends by 47%. The SEM and PLM results confirm that HDPE are distributed in PP in the form of particles. The combination of HDPE is able to decrease the size of PP’s spherulites, and in turn significantly heightens the impact strength of PP matrices. FTIR results show that the combination of HDPE is not correlated with the chemical structure of PP, which indicates that the polyblends are formed via a physical blending. Finally, DSC and XRD results show that the combination of HDPE can effectively improve the crystallization properties of PP without changing the crystalline structure of PP. The PP/HDPE polyblends prepared by this study have a low production cost and efficient processing. Therefore, common applications can be expected.
